# Extrasystoles for fluid responsiveness prediction in critically ill patients

**DOI:** 10.1186/s40560-018-0324-6

**Published:** 2018-08-22

**Authors:** Simon Tilma Vistisen, Martin Buhl Krog, Thomas Elkmann, Mikael Fink Vallentin, Thomas W. L. Scheeren, Christoffer Sølling

**Affiliations:** 1 Research Centre for Emergency Medicine, Institute of Clinical Medicine, Aarhus University, Palle Juul-Jensens Boulevard 99, 8200 Aarhus N, Denmark; 2 Department of Anesthesiology and Intensive Care, Aarhus University Hospitals, Aarhus, Denmark; 3 University Medical Center Groningen, Department of Anesthesiology, University of Groningen, Groningen, the Netherlands; 4 Department of Anesthesiology and Intensive Care, Viborg Regional Hospital, Viborg, Denmark

**Keywords:** Hemodynamic monitoring, Fluid responsiveness, Extrasystole, Ectopic beat, Stroke volume, Cardiac output

## Abstract

**Background:**

Fluid responsiveness prediction with continuously available monitoring is an unsettled matter for the vast majority of critically ill patients, and development of new and reliable methods is desired. We hypothesized that the post-ectopic beat, which is associated with increased preload, could be analyzed in relation to preceding sinus beats and that the change in cardiac performance (e.g., systolic blood pressure) at the post-ectopic beat could predict fluid responsiveness.

**Methods:**

Critically ill patients were observed when scheduled for a 500-ml volume expansion. The 30-min ECG prior to volume expansion was analyzed for the occurrence of extrasystoles. Classification variables were defined as the change in a variable (e.g., systolic blood pressure or pre-ejection period) from the median of ten preceding sinus beats to extrasystolic post-ectopic beat. A stroke volume increase > 10% following volume expansion defined fluid responsiveness.

**Results:**

Twenty-six patients were included. The change in systolic blood pressure predicted fluid responsiveness with receiver operating characteristic (ROC) area 0.79 (CI [0.52:1.00]), specificity 100%, sensitivity 67%, positive predictive value 100%, and negative predictive value 91% (threshold: 5%). The change in pre-ejection period predicted fluid responsiveness with ROC area 0.74 (CI [0.53:0.94]), specificity 78%, sensitivity 67%, positive predictive value 50%, and negative predictive value 88% (threshold 7.5 ms).

**Conclusions:**

Based on standard critical care monitoring, analysis of the extrasystolic post-ectopic beat predicts fluid responsiveness in critical care patients with good accuracy. The presented results are considered preliminary proof-of-concept results, and further validation is needed to confirm these preliminary findings.

**Electronic supplementary material:**

The online version of this article (10.1186/s40560-018-0324-6) contains supplementary material, which is available to authorized users.

## Background

Fluid responsiveness prediction remains an unresolved issue for most ICU patients. Ventilator-induced dynamic variables, such as pulse pressure variation (PPV) or stroke volume variation (SVV), are only optimally reliable in 2–3% of admitted ICU patients [1], and passive leg raising (PLR) is time-consuming to perform and requires cardiac output monitoring to give optimal prediction [2]. Still, when used within their limitations, these two monitoring concepts are by far the best validated methods for fluid responsiveness prediction across various patient populations in the ICU setting [3–6]. However, sparsely reported data from the largest PLR study to date [7] indicate that PLR may have a slightly reduced predictive power [8] compared with the estimates from a meta-analysis [4]. The two techniques are at the core of fluid responsiveness research, and both concepts rely on the assumption that a standardized preload fluctuation will induce large fluctuations in, e.g., stroke volume (SV) or pulse pressure (PP) in fluid responders compared with non-responders. So, in the search for equally reliable but easier and more applicable fluid responsiveness methods for ICU patients, the preload fluctuation concept is likely the way forward.

Recently, we suggested that an extrasystole could be considered as a preload varying mechanism [9, 10]. While the ectopic beat itself is a poor heartbeat due to its premature nature, the post-ectopic beat is associated with an increased preload due to the compensatory pause and probably also due to a poor ejection at the ectopic beat [11, 12]. The post-ectopic beat is otherwise a sinus beat, and therefore, the post-ectopic beat is likely to elucidate the effect of increased preload and can be seen as a one-heartbeat reversible fluid challenge to the heart.

In a recent study, we showed that the post-ectopic change in the systolic blood pressure (SBP) and pre-ejection period (PEP) predicted fluid responsiveness with good accuracy in postoperative cardiac surgery patients [10] (both had an area under the receiver operating characteristic (ROC) curve of 0.81). However, post-cardiac surgery patients in their recovery phase are clinically very different from critically ill patients in the ICU. In the present study, we investigated the post-ectopic beat characteristics’ ability to predict fluid responsiveness in critically ill patients admitted to a more general ICU and scheduled for a volume expansion.

## Methods

The Regional Ethics Committee, Central Region, Denmark, and the Danish Health and Medicines Authority considered the study design observational. The study was approved by the Danish Data Agency (1-16-02-83-15), and it was registered at ClinicalTrials.gov (NCT02520037). Data was collected from June 2015 to September 2016.

### Patients and inclusion/exclusion criteria

Critically ill patients aged 18 years or more admitted to our intensive care unit were observed if a volume expansion of 500 ml crystalloid was scheduled to be infused within 30 min or less on clinical reasons. Patients had been assessed with ultrasonography (Focus Assessed Transthoracic Echocardiography) as part of the clinical decision-making of prescribing a volume expansion (including crude assessment of left ventricular function by eyeballing). Patients with arrhythmia precluding the use of the extrasystoles method (i.e., atrial fibrillation, trigemini, or obscure pacing rhythm) were not observed. The study time frame was up to 30 min prior to volume expansion until 5 min after volume expansion. Any changes made in hemodynamically relevant treatments during this period excluded the observation, i.e., any changes in vasopressor, inotropic, analgesic or anesthetic infusion rates, changes in positive end-expiratory pressure (PEEP), and body position in the bed.

Sepsis was defined according to the previous criteria [13] because the study was initiated prior to the publication of the new sepsis criteria [14].

### Data acquisition

The 30-min ECG and arterial pressure waveforms prior to volume expansion were extracted using Philips Research Data Export software. These waveforms were sampled at 125 Hz and subsequently upsampled to 1000 Hz as previously described [15]. Stroke volume (SV) before and after fluid infusion was measured with a non-invasive bioreactance-based cardiac output monitor (NICOM®, Cheetah Medical, Newton Center, MA, USA). The 5-min average of ten consecutive SV measurements before fluid infusion defined the baseline SV, and the 5-min SV average immediately following the fluid infusion defined the SV after fluid infusion. No hemodynamic changes were made during SV measurements. Other hemodynamic variables, ventilator settings, and blood gases were manually registered.

### Detection and eligibility of extrasystoles

Detection of extrasystoles was done semi-automatically by custom-made R spike detection algorithms in Matlab (Version 2014a, Mathworks Inc., MA, USA). Potential extrasystoles were visually checked for eligibility defined by being preceded by ten sinus beats (representing baseline heartbeats) and the coupling interval being 80% or less than the preceding sinus beat (resulting in a relevant preload change [9]). For each eligible extrasystole, the fluctuation in SBP, PEP, PP, and maximal slope of systolic upstroke (dP/dt) was derived from the arterial blood pressure curve. The fluctuation was defined as the difference from the post-ectopic beat and the median value for the ten preceding sinus beats as previously described (supplemental material in [10]). Differences were calculated as both relative and absolute changes for SBP, PEP, PP, and dP/dt and referred to as, for example, SBP_rel_ and PEP_abs_.

In case of more than one eligible extrasystole in the 30-min period before volume expansion, the derived variables for all extrasystoles were averaged (median) to represent a single number for an extrasystolic change in that variable per patient. The ten most recent extrasystoles prior to the volume expansion were used if there were more than ten extrasystoles in the 30-min period prior to the volume expansion.

### Classification, data analysis, and statistics

Fluid responsiveness was initially defined as a 15% or more increase in SV, and results for this threshold is reported, but the limit was reduced to 10% upon data collection for statistical purposes due to only a few cases of fluid responsiveness to classify at the initial fluid response threshold of 15%.

Paired *t* test was used to compare hemodynamic variables prior to and after volume expansion. PPV was calculated for patients meeting the ventilator setting criteria for PPV. Central venous pressure is not standard monitoring in our ICU but was collected when available. We did not investigate additional variables for fluid responsiveness prediction since these were not readily available. All waveform data signal processing were done with Matlab. ROC area statistics are reported as “estimate [confidence interval]” along with optimal sensitivity and specificity measures according to the Youden index. The combined classification results from this and our previous clinical study in post-cardiac surgery patients are also reported. All statistical tests were performed with R (R studio, version 3.2.3 using package “pROC” for ROC statistics with the DeLong method used for ROC area confidence intervals). Spearman correlation is reported. Paired *t* test was used to compare the hemodynamic variables before and after fluid infusion. Student’s *t* test was used to compare the hemodynamic variables between responders and non-responders. Based on previous data, we calculated the sample size. Using a significance level of 0.05 and power of 0.8 and assuming equal numbers of fluid responders and non-responders, we needed 23 patients with extrasystoles to provide a ROC area significantly different from 0.5. Assuming that half of the patients had one or more extrasystoles in the observation window, we aimed at 46 patients.

## Results

**Fig. 1:**
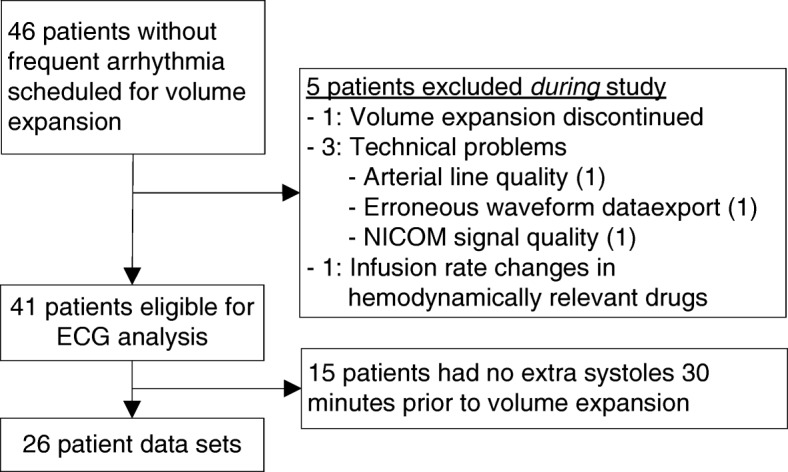
Inclusion of patients

Patient inclusion is shown in Fig. 1. Demographic and clinical data are presented in Table 1 for the included patients with extrasystoles. Twenty-six out of 41 (63%) patients eligible for ECG analysis had at least one eligible extrasystole prior to the scheduled fluid challenge, and they were included in the fluid responsiveness prediction analysis. Sixteen of the included patients were intubated but only three patients were ventilated in controlled mode and fully adapted to the ventilator, and tidal volumes for these patients were 6.4, 6.7, and 8.8 ml/kg predicted body weight. CVP was monitored in two patients. Therefore, the classification performance of PPV and CVP is not reported.

**Table 1:** Demographic and clinical characteristics of patients

*n* = 26		
Age, years	70.1 (11.2)	
Gender, male/female	9/17	
Height, cm	168 (7.9)	
Weight, kg	65.7 (16.7)	
SOFA score	7.2 (3.2)	
Lactate	2.1 (1.5)	
Primary diagnosis		
-Severe sepsis	4	
-GI origin	2	
-Origin not confirmed	2	
-Septic shock	14	
-GI origin	5	
-Pneumonia	5	
-Origin not confirmed	4	
-Severe hypotension after major orthopedic surgery	2	
-Cerebral event	2	
-GI bleeding	1	
-Chronic pancreatitis	1	
-Rhabdomyolysis	1	
-Acute liver failure	1	
Vasoactive/inotropic drugs		
-Norepinephrine	17	
-Dobutamine	1	
Hemodynamics	Before fluids	After fluids
HR, min^−1^	90 (18)	88 (19)
MAP, mmHg	73 (15)	82 (14)*
SV, ml	72 (23)	71 (22)

*GI* gastro-intestinal, *HR* heart rate, *MAP* mean arterial pressure, *SV* stroke volume

*p < 0.00001

In two cases, the post-ectopic change in PEP and PP could not be calculated for technical reasons (waveform “cut” near the diastolic pressure level), leaving 24 datasets for these variables.

Six (23%) patients responded to fluids with an increase in SV of at least 10%.

**Fig. 2:**
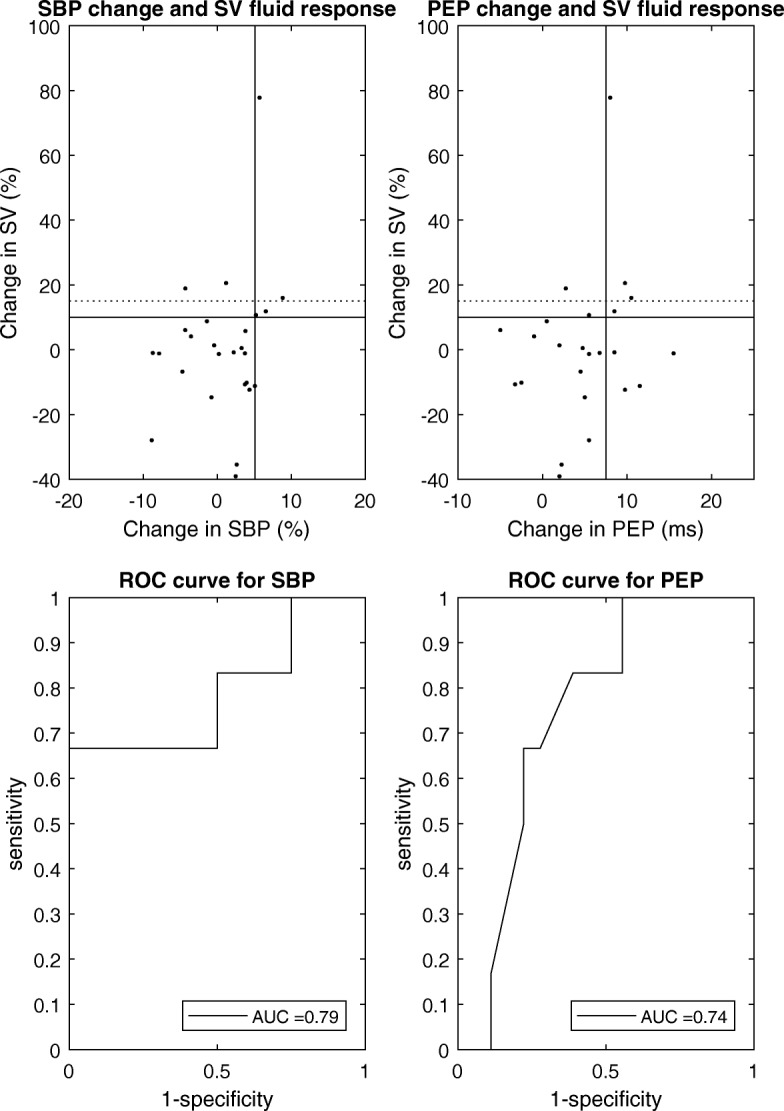
Fluid responsiveness prediction characteristics of the systolic blood pressure (SBP) and pre-ejection period (PEP) change at post-ectopic beat. ROC curves are shown for the 10% increase in stroke volume (SV) following fluid infusion

Classification characteristics of the four variables investigated during post-ectopic beats are shown in Table 2 and Fig. 2. SBP_rel_ predicted fluid responsiveness with ROC area of 0.79, with sensitivity of 67%, specificity of 100%, negative predictive value of 91%, and positive predictive value of 100%. PEP_abs_ predicted fluid responsiveness with ROC area of 0.74, with sensitivity of 67%, specificity of 78%, negative predictive value of 88%, and positive predictive value of 50%. Table 3 presents the baseline values and absolute post-ectopic changes of SBP, PEP, PP, and dP/dt for fluid responders and fluid non-responders. The correlation between variables presented in Fig. 2 did not reach a statistical significance (rho = 0.19, *p* = 0.36, for SBP_rel_ and rho = 0.14, *p* = 0.50, for PEP_abs_). The present study’s data combined with previously reported clinical data (SBP_rel_ and PEP_abs_) is shown in Additional file 1: Figure S1, where AUC point estimates ranged from 0.72 to 0.81.

**Table 2:** Classification characteristics of post-ectopic changes in variables

	Prediction at 10% SV change threshold				Prediction at 15% SV change threshold			
Variable	ROC curve area	Spec (%)	Sens (%)	Threshold	ROC curve area	Spec (%)	Sens (%)	Threshold
SBP_abs_	0.78 [0.54; 1]	90	67	5.5 mmHg	0.66 [0.33; 0.99]	82	50	5.5 mmHg
PEP_abs_	0.74 [0.53; 0.95]	78	67	7.5 ms	0.73 [0.47; 1]	75	75	7.5 ms
PP_abs_	0.77 [0.52; 1]	78	83	8.46 mmHg	0.73 [0.36; 1]	75	75	8.46 mmHg
dP/dt_abs_	0.76 [0.53; 0.99]	85	67	0.195 mmHg/s^2^	0.64 [0.33; 0.94]	32	100	0.030 mmHg/s^2^
SBP_rel_	0.79 [0.52; 1]	100	67	5%	0.66 [0.27; 1]	95	50	5.5%
PEP_rel_	0.70 [0.50; 0.90]	65	83	2.5%	0.69 [0.44; 0.95]	68	75	3%
PP_rel_	0.70 [0.48; 0.93]	67	83	15%	0.71 [0.41; 1]	80	75	15%
dP/dt_rel_	0.68 [0.39; 98]	70	83	14%	0.68 [0.39; 0.98]	64	75	14%

*SBP* systolic blood pressure, *PEP* pre-ejection period, *PP* pulse pressure, *dP/dt* maximal systolic upstroke, *SV* stroke volume, *ROC* receiver operating characteristic, *Spec* specificity, *Sens* sensitivity, *rel* post-ectopic change calculated on relative scale, *abs* post-ectopic change calculated on absolute scale

**Table 3:** Hemodynamic characteristics of sinus beats prior to ectopy and the post-ectopic beat for fluid non-responders and fluid responders

	Non-responders	Responders	*p* value
Baseline SBP (mmHg)	116 (21)	125 (35)	0.46
Post-ectopic SBP change (mmHg)	− 0.1 (5.6)	5.9 (6.6)	0.04
Baseline PEP (ms)	212 (39)	212 (34)	0.99
Post-ectopic PEP change (ms)	4.0 (5.3)	7.5 (2.9)	0.14
Baseline PP (mmHg)	58 (21)	65 (22)	0.51
Post-ectopic PP change (mmHg)	4.0 (6.7)	10.6 (7.0)	0.05
Baseline dP/dt (ms)	0.88 (0.47)	0.94 (0.39)	0.77
Post-ectopic dP/dt change (mmHg/ms)	0.08 (0.11)	0.16 (0.08)	0.10

*SBP* systolic blood pressure, *PEP* pre-ejection period, *PP* pulse pressure

## Discussion

In the present study, we investigated the post-ectopic beat characteristics’ ability to predict fluid responsiveness in critically ill patients. Four out of six fluid responders (increasing SV by 10% or more) had a post-ectopic SBP_rel_ change exceeding 5%, whereas none of the 20 non-responders had post-ectopic SBP changes exceeding that level, resulting in estimated sensitivity and specificity of 67% and 100%, respectively. As such, our study confirms our previous clinical findings that a post-ectopic SBP change of more than 5% appears a safe threshold for predicting a significant increase in SV after fluid loading, which, however, is on the expense of a sensitivity level of around 70% [10].

Extrasystoles were available in 63% of the observed non-atrial fibrillation patients in this study. In our previous clinical study in post-cardiac surgery patients, we found a similar incidence (61%). Still, both of these studies’ samples are considered convenience samples, but these figures are in alignment with a more detailed extrasystolic occurrence analysis (paper under review). The detection of extrasystoles was done semi-automatically in this study because the monitor’s automatic annotation of hearts beat was not available. Eventually, detection of extrasystoles should be automated by existing monitor software as well as the interbeat calculation of SPB, PP, etc. These detections are already done in existing monitoring, but not combined. The steady-state period of 30 min observation for extrasystoles is obviously not suggested as a waiting time for clinicians, rather the idea is that clinicians facing hemodynamically unstable patients could look back in time and supplement their intervention decision with the arterial waveform characteristics of recent extrasystoles (< 30 min). This is (as estimated) possible in more than half of non-atrial fibrillation patients. While not directly comparable, this stands in contrast to 2–3% of patients where PPV is reliable in the ICU. Still, there is a large group of patients (here, estimated 37%), where extrasystoles had not spontaneously occurred prior to volume expansion. In these cases, the extrasystole method is not applicable. It is up to the clinicians to decide whether the glass is half full or half empty with this extrasystolic occurrence. We think of it as half full because the information held in extrasystoles is, in principle, freely and readily available in half of the ICU patients monitored with ECG and invasive arterial pressure. Obviously, however, post-ectopic beat characteristics need to be calculated by monitors since eyeballing small changes in SBP is difficult and even impossible for PEP changes. For the method to work, these calculations have to be automated by monitors.

Our study is associated with some limitations that should be taken into account when interpreting the data. In this observational study, we included a heterogenic patient population. The study was small (*n* = 26), and we encountered few responders to classify with our method. Therefore, we have to pay discrete and meticulous caution for the data interpretation. The low number of responders leads to the choice of reducing the otherwise prospectively defined SV response threshold from 15 to 10%, which is a suboptimal modification to the registered study design. Still, combining the study results with previously reported clinical data (Additional file 1: Figure S1) is supporting the conclusion that the extrasystolic method is predicting fluid responsiveness in ICU patients with good accuracy. However, while Additional file 1: Figure S1 is summarizing the classification accuracy of the existing data for the extrasystoles method, it has to be kept in mind that the combined data originates from two different ICU patient populations, namely postoperative cardiac surgery patients [10] and in this study more regular ICU patients not including any post-cardiac surgery patients.

While not anticipating the low number of responders, we speculate that it could be related to the fact that most of the patients could be described as being in their “optimization/stabilization” phase, where a massive increase in stroke volume upon fluid loading is less likely as opposed to, for example, the early “rescue” hours in sepsis [16], where a future study preferentially could be conducted. Infusion time (30 min) might also be considered long, but patients increased their mean arterial pressure (MAP) significantly to the fluid challenge as opposed to their SV (Table 1), so from some clinical viewpoints, more than six patients responded to fluids. In addition, our SV monitoring technique, NICOM®, may also be an explanation for the few fluid responders. CO measurements fluctuate as a consequence of both interventions (e.g., fluids), physiologic variation over time and measurement error. NICOM® bias was reported to be + 4.1% ± 11.3%, and sensitivity and specificity for significant directional changes were both 93% [17]. In another study, the measured SV response on fluid loading as measured by NICOM® was also modest (4 out of 48 patients increased NICOM®-derived SV by 15% or more upon volume expansion) [18]. The validity of NICOM® to detect these changes has therefore been debated [17, 18], and the use of bioreactance techniques for measuring CO and/or SV in the everyday *clinical setting* may not be ready for expert panel endorsement [19], because absolute values are of importance. Still, NICOM®-derived SV is a reasonable outcome measure for fluid responsiveness *research* [17, 20]. Finally, we did not compare the extrasystolic method with other fluid responsiveness predictors such as PLR and CVP. PLR, despite the need for intervention and reliable, fast responding SV measurements, is currently the most applicable method in ICUs. Additionally, PLR has reportedly a very high accuracy of predicting fluid responsiveness as estimated by systematic reviews [3–5]. However, the largest and probably best-conducted PLR study to date [7] is revealing somewhat lower prediction accuracy (sensitivity and specificity *estimated* at 84% and 62%, respectively [8]). Surprisingly, the authors of that study did not wish to provide exact classification statistics in their study [7] despite data for it was undoubtedly available and despite the authors were encouraged to [8]. Consequently, and since we did not directly compare with PLR in this study, it becomes difficult to discuss what classification accuracy should be considered acceptable for competing methods like the one presented. It is up to the readers to judge, but we think that the available clinical data shows that post-ectopic beat characteristics could supplement the decision of whether or not to give a fluid infusion in ICU patients since the method does not rely on an intervention. However, the extrasystolic morphologic configuration in ABP should not be the sole reason for administering or not administering fluids since the classification accuracy is not excellent and since a lot of other clinical factors should be taken into account in the decision of administering fluids, including risk of side effects to fluids, which fluid responsiveness methods generally cannot assess.

There are fundamental differences between supraventricular and ventricular extrasystoles. Particularly, the compensatory pause is different (longer) for ventricular extrasystoles compared with supraventricular extrasystoles. However, in the clinical studies conducted so far, the post-ectopic changes encountered in, for example, SBP and PEP are not different when comparing the supraventricular and ventricular extrasystoles and they appear equally predictive of fluid responsiveness. This was the reason why we included both types of extrasystoles in the study. It has been hypothesized that both preload and contractility of the heart are altered during the ectopic activity, contributing to the compensatory mechanism of reduced stroke volume at the ectopic beat [11]. While left ventricular end-diastolic pressure is always increased at the post-ectopic beat compared with the preceding sinus beats [11], contractility may also contribute to the observed changes in cardiac performance at the post-ectopic beat [11], and it has been hypothesized that calcium derangements occur at the post-ectopic beat. A contractility altering mechanism could explain why the simple post-ectopic evaluation of blood pressure characteristics does not excellently predict fluid responsiveness with a ROC AUC exceeding 0.90. Still, in vivo studies testing the contractility derangements have so far not investigated this mechanism under various preload conditions in the intact heart, so it remains unclear how much a contractility altering effect contributes to the contraction at the post-ectopic beat compared with the preload altering the effect.

Respiration, particularly mechanical ventilation, influences the beat-to-beat level of hemodynamic variables such as SBP. In our data analyses, we averaged out this effect as we calculated a median value from ten preceding heartbeats. However, the exact occurrence of an extrasystole within a respiratory cycle may influence the value of SBP at the post-ectopic beat.

In our study, we did not evaluate the exclusion of frequent arrhythmia (predominantly atrial fibrillation), but the overall occurrence of atrial fibrillation in medical and non-cardiac surgical adult intensive care unit patients is estimated at 10.5% [21].

The magnitude of the post-ectopic PEP_abs_ change was markedly different between this and our previous study as reflected in the optimal classification thresholds (7.5 ms in this study and 19 ms in our previous study [10]). We do not have any reason to believe that the difference could be related to the monitoring technology, since pressure transducers, monitors, the data extraction, and the algorithms to derive PEP were the same across studies. It must be related to the patient category and/or how patients were treated. Looking detailed into this and the previous study’s data, indeed, there appears to be an explanation: baseline PEP (at sinus beats and according to our definition) was 253 ms (SD: 33 ms) for cardiac surgery patients in their first postoperative hours, whereas PEP was 212 ms (SD 37 ms) in the present study’s patients. The vascular transit time (time from pressure upstroke in the aorta to pressure upstroke in the radial artery) is around 80–100 ms and probably does not vary much across our populations. Since the post-ectopic PEP change is defined by us as an absolute value (in ms and not percent), this physiologic difference between the two patient populations may—in combination with patient characteristics and the low number of fluid responders in the present study—explain the observed difference in the magnitude of absolute post-ectopic PEP changes. No matter the underlying explanation for the differences in post-ectopic PEP changes, which we speculate to be related to cardiac function and/or cardiac medication differences, it should be noted that this variable has to be investigated more thoroughly in various patient categories before it can be suggested to be used for fluid responsiveness prediction.

## Conclusion

This study’s data further supports the notion that extrasystoles may be useful for fluid responsiveness prediction, but we have not encountered excellent prediction with the extrasystoles method. Due to the limitations of our study along with the amount of available clinical data on extrasystoles’ ability to predict fluid responsiveness, we still consider the data at hand preliminary proof-of-concept results and we would be cautious and not yet recommend the method for clinical use before more validation studies point in the same direction as the currently available data, preferably carried out by other research groups. So far, however, a 5% post-ectopic increase in systolic blood pressure appears a consistent indicator of a positive fluid response, and this observation may be useful in situations where other reliable fluid responsiveness monitoring is not available.

## Additional file

Additional file 1: **Figure S1.** Data combining previous clinical data with the present study’s data. Dot markers constitute data from this study. Star markers constitute data from the previous study. Middle panels are ROC curves for predicting fluid responsiveness at the 15% stroke volume (SV) increase threshold (dashed horizontal lines in upper panels), whereas the lower panels are ROC curves for the 10% SV increase threshold (full horizontal lines in upper panels). (PDF 8 kb)
