# Supplementary figures and images for: Extrasystoles for fluid responsiveness prediction in critically ill patients
Source: J Intensive Care. 2018 Aug 22;6:52. doi: 10.1186/s40560-018-0324-6 (PMC6103985; doi:10.1186/s40560-018-0324-6)

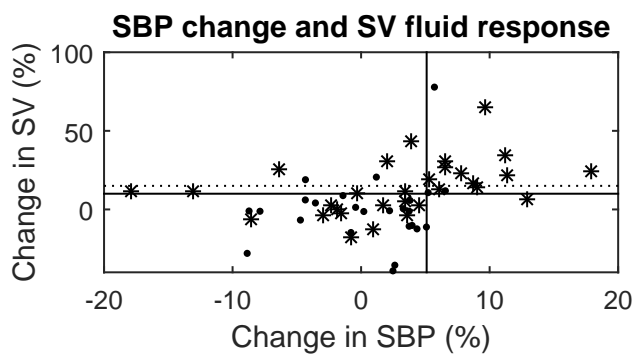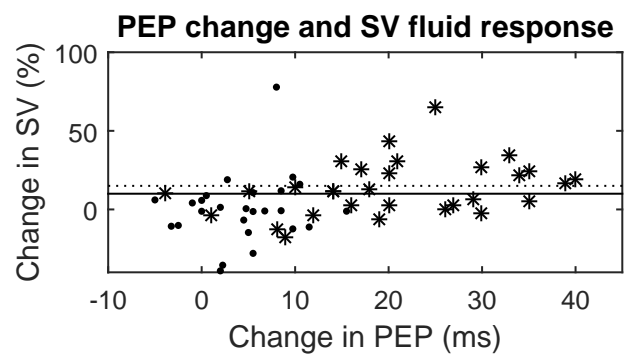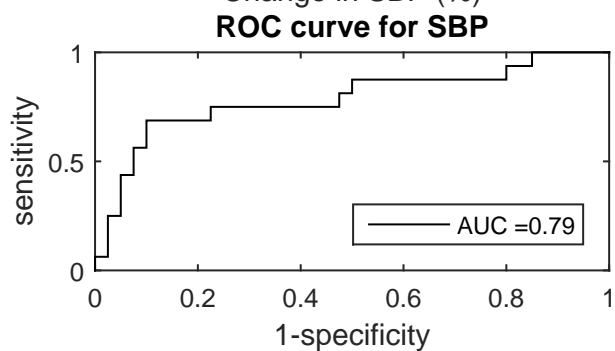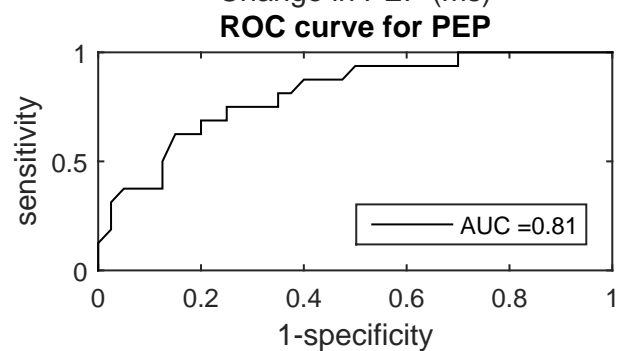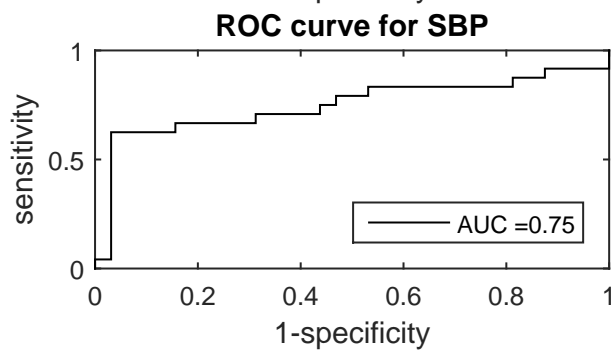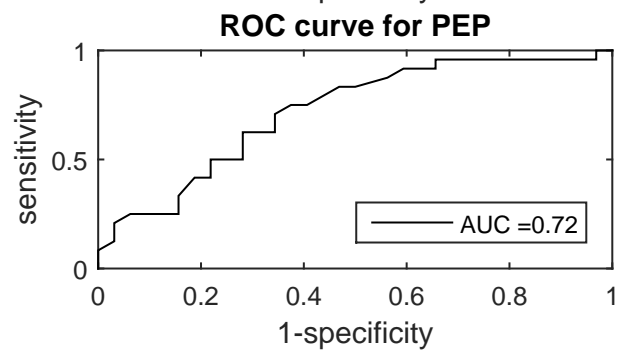

Supplement: Supplementary file 1 — Figure S1. Data combining previous clinical data with the present study’s data. Dot markers constitute data from this study. Star markers constitute data from the previous study. Middle panels are ROC curves for predicting fluid responsiveness at the 15% stroke volume (SV) increase threshold (dashed horizontal lines in upper panels), whereas the lower panels are ROC curves for the 10% SV increase threshold (full horizontal lines in upper panels). (PDF 8 kb) [file 40560_2018_324_MOESM1_ESM.pdf]
